# *Allobaculum* Involves in the Modulation of Intestinal ANGPTLT4 Expression in Mice Treated by High-Fat Diet

**DOI:** 10.3389/fnut.2021.690138

**Published:** 2021-05-19

**Authors:** Zibin Zheng, Wentao Lyu, Ying Ren, Xiaoqiong Li, Shenjun Zhao, Hua Yang, Yingping Xiao

**Affiliations:** ^1^State Key Laboratory for Managing Biotic and Chemical Threats to the Quality and Safety of Agro-Products, Institute of Agro-Product Safety and Nutrition, Zhejiang Academy of Agricultural Sciences, Hangzhou, China; ^2^Hubei Key Laboratory of Animal Nutrition and Feed Science, Wuhan Polytechnic University, Wuhan, China

**Keywords:** gut microbiota, ANGPTL4, fat deposition, high-fat diet, mice

## Abstract

Increasing studies have shown that obesity is the primary cause of cardiovascular diseases, non-alcoholic fatty liver diseases, type 2 diabetes, and a variety of cancers. The dysfunction of gut microbiota was proved to result in obesity. Recent research indicated ANGPTL4 was a key regulator in lipid metabolism and a circulating medium for gut microbiota and fat deposition. The present study was conducted to investigate the alteration of gut microbiota and ANGPTL4 expression in the gastrointestinal tract of mice treated by the high-fat diet. Ten C57BL/6J mice were randomly allocated to two groups and fed with a high-fat diet (HFD) containing 60% fat or a normal-fat diet (Control) containing 10% fat. The segments of ileum and colon were collected for the determination of ANGPTL4 expression by RT-qPCR and immunohistochemical analysis while the ileal and colonic contents were collected for 16S rRNA gene sequencing. The results showed HFD significantly increased mice body weight, epididymal fat weight, perirenal fat weight, liver weight, and the lipid content in the liver (*P* < 0.05). The relative expression of ANGPTL4 and the ANGPTL4-positive cells in the ileum and colon of mice was significantly increased by HFD treatment. Furthermore, 16S rRNA gene sequencing of the ileal and colonic microbiota suggested that HFD treatment changed the composition of the gut microbiota. The ratio of Firmicutes to Bacteroidetes and the abundance of *Allobaculum* was significantly higher in the HFD group than in the Control group while the abundance of *Adlercreutzia, Bifidobacterium, Prevotellaceae UCG-001*, and *Ruminococcus* was significantly decreased. Interestingly, the abundance of *Allobaculum* was positively correlated with the expression of ANGPTL4. These findings provide a theoretical foundation for the development of strategies to control the obesity and related diseases by the regulation of ANGPTL4 and gut microbiota.

## Introduction

The epidemic rise in obesity has chronically challenged human health, performance and quality of life with affecting more than 2 billion people in the world and being related to diabetes, cancers and cardiovascular diseases (1, 2). Gut microbiota is composed of numerous bacteria that contribute to nutrition absorption and energy homeostasis (3, 4). Increasing evidences indicate that gut microbiota directly participates in obesity and many other metabolic diseases (4, 5). A recent study have shown that obesity could be induced by the high-fat diet (HFD) (6). Meanwhile, the dysregulation in the composition and metabolic functions of gut microbiota would promote the development of obesity (7). The microbiota transplantation from girls with or without obesity to mice showed a close relationship between gut microbiota and obesity (8). Furthermore, the significant higher abundance ratio of Firmicutes to Bacteroidetes is generally regarded as a marker signal of obesity (9). Therefore, gut microbiota is closely related to host lipid metabolism, the disorder of which alters the composition of gut microbiota. The dysfunctional gut microbiota would further affects the host lipid metabolism in turn (10).

Angiogenin-like protein 4 (ANGPTL4), also known as a fasting induction factor (FIAF), plays an important role in lipid deposition by inhibiting lipoprotein lipase (LPL) to regulate lipid metabolism (11, 12). ANGPTL4 can be secreted in intestines, adipose tissue, liver, skeletal muscle, heart and other tissues, and is subsequently cleaved into N-terminal and C-terminal fragments. The N-terminal of ANGPTL4 acts as a LPL inhibitor (13, 14). LPL is transported by the GPIHBP1 protein to the lumen side of capillary endothelial cells and catalyzes the hydrolysis of triglycerides (TG) into fatty acids. This process allows lipids transported from the circulation into skeletal muscle, heart and adipose tissue after absorption (15, 16). In addition, ANGPTL4 is an endogenous inhibitor for intestinal fat digestive enzymes, especially pancreatic lipase, to prevent excessive fatty acids intake and lipid overload in intestinal cells (17, 18). Furthermore, ANGPTL4 has been considered as a circulating mediator between gut microbiota and fat storage (19). The germ-free (GF) mice with a normal microbiota harvested from the cecum of conventionally raised mice could improve the TG storage in fat cells with inhibition of ANGPTL4 expression in gut, suggesting gut microbiota might promote fat deposition by modulating ANGPTL4 expression (19). The expression of lipogenic genes in the abdominal fat of mice receiving the fecal microbiota of Jinhua pigs (obese) was higher than that in the mice receiving the fecal microbiota of Landrace pigs (lean) with reduction of ANGPTL4 expression in the gastrointestinal tract (20). Therefore, the ANGPTL4 expression might be one of the key regulators for obesity induced by the dysfunction of gut microbiota.

ANGPTL4 acts as a circulating medium for the gut microbiota and fat deposition in the body, so it is of great significance to explore how the gut microbiota affects the expression of ANGPTL4. However, the research on the regulation of ANGPTL4 expression by gut microbiota in obesity is limited. Accordingly, the objective of the present study is to explore the relationship among the gut microbiota structure, the ANGPTL4 expression and fat deposition.

## Materials and Methods

### Animals and Sampling

Ten specific pathogen-free (SPF) C57BL/6J male mice weaned at the age of 28 days were purchased from GemPharmatech Co., Ltd (Nanjing, China). The mice were raised in cages at 25 ± 2°C for 12 h light/dark cycles with free access to water and mouse chow. After acclimatization for 1 week, the mice were weighed and randomly divided into Control group and high-fat diet (HFD) group. Mice in the Control and HFD groups were fed with a commercial standard diet with 10% fat content and the standard diet supplemented with 60% fat, respectively for 12 weeks (21, 22). At the end of 12-week study, all mice were weighed individually and sacrificed by decapitation following a CO_2_ stun. The epididymal fat and perirenal fat of each mice were isolated and weighed. The ileal and colonic contents were collected and stored at −20°C until DNA isolation and 16S rRNA gene sequencing. Segments of liver, ileum and colon were collected and fixed in 4% paraformaldehyde for further analysis. Ileum and colon segments were isolated, rinsed with 0.9% NaCl, immediately frozen in liquid nitrogen, and stored at −80°C until RNA extraction.

### DNA Extraction, Sequencing, and Data Analysis

Microbial genomic DNA was extracted from ileal and colonic contents using QIAamp DNA Stool Mini Kit (QIAGEN, Valencia, CA, US). The V4~V5 region of bacterial 16S rRNA gene was amplified from each genomic DNA sample by using the barcode-fusion primers 515F and 907R. Sequencing libraries were then constructed using TruSeq DNA PCR-Free Library Preparation Kit (Illumina) and sequenced on an Illumina HiSeq platform at Mingke Biotechnology (Hangzhou) Co., Ltd. The sequencing data were analyzed using QIIME software package (23). The non-repeating sequences were analyzed by operational classification unit (OTU) with 97% similarity. Species matching was performed for all representative sequences of OTU using RPD databases (24). Pie charts were generated to show taxa distribution at the phylum and genus levels. Principal coordinate analysis (PCoA) was performed to analyze the beta diversity. For further identify the specific genera related to fat deposition, this study also analyzed the raw data of gut microbiota combining with other two similar studies on mice fed with high-fat diets (DDBJ Accession Number: PRJDB7523) (25) (NCBI Accession Number: SRP113647) (26).

### RNA Extraction and Real-Time Quantitative PCR

Total RNA was isolated using TRIzol^®^Plus RNA Purification Kit (Invitrogen) and RNase-Free DNase Set (Qiagen) followed by reverse transcription using the SuperScript^™^ III First-Strand Synthesis SuperMix (Invitrogen) strictly according to the manufacturer’s instructions. Real-time qPCR was performed in triplicate on an ABI Prism 7700 Sequence Detector system (Applied Biosystems, Foster City, CA, USA) using an annealing temperature of 63°C and gene-specific primers listed in Table 1. The data were normalized to GAPDH or 18S rRNA and calculated by 2^−ΔΔCT^ method (27).

**Table 1:** Primers used in the RT-qPCR analysis.

**Gene**	**Genbank**	**Primer sequences (5**^**′**^**-3**^**′**^**)**	**Size (bp)**
	**accession**		
Pig GAPDH	NM_001206359.1	CCAGGGCTGCTTTTAACTCTG	104
		GTGGGTGGAATCATACTGGAACAT	
Pig ANGPTL4	NM_001038644.1	GACTGCCAAGAGCTGTTTGAAGA	126
		CTGAATTACAGTCCAGCCTCCAT	

### Histological Staining and Immunohistochemistry

The liver egments were fixed in 4% paraformaldehyde for 1 h at room temperature, cryoprotected in 20% sucrose at 4°C overnight, and embedded in OCT. The prepared series of 12-μm cryosections were stained with Oil Red O (Sigma-Aldrich, St. Louis, MO, United States).

The ileal and colonic sections were deparaffinized and microwaved in sodium citrate buffer for antigen retrieval. After being rinsed with phosphate buffered saline (PBS), slides were incubated with PBS containing normal goat serum. After blocking, sections were incubated with primary antibody (proteintech ANGPTL4 18374-1-AP) at 4°C overnight. After wash with PBS and incubation with secondary antibody for 50 min at room temperature, slides were rinsed with PBS for three times and added with a few drops of chromogenic substrate DAB with incubation for 15 min. Finally, slides were rinsed in water and counterstained using hematoxylin solution for picture caption.

### Statistical Analysis

All statistical analyses were performed by SPSS 23.0 (IBM, New York, NY, United States) using unpaired two-tailed Student’s *t*-test (20). Data are presented as the mean ± SEM. Results were considered significant when *P* < 0.05.

## Result

### Obesity Induced by HFD

**Figure 1:**
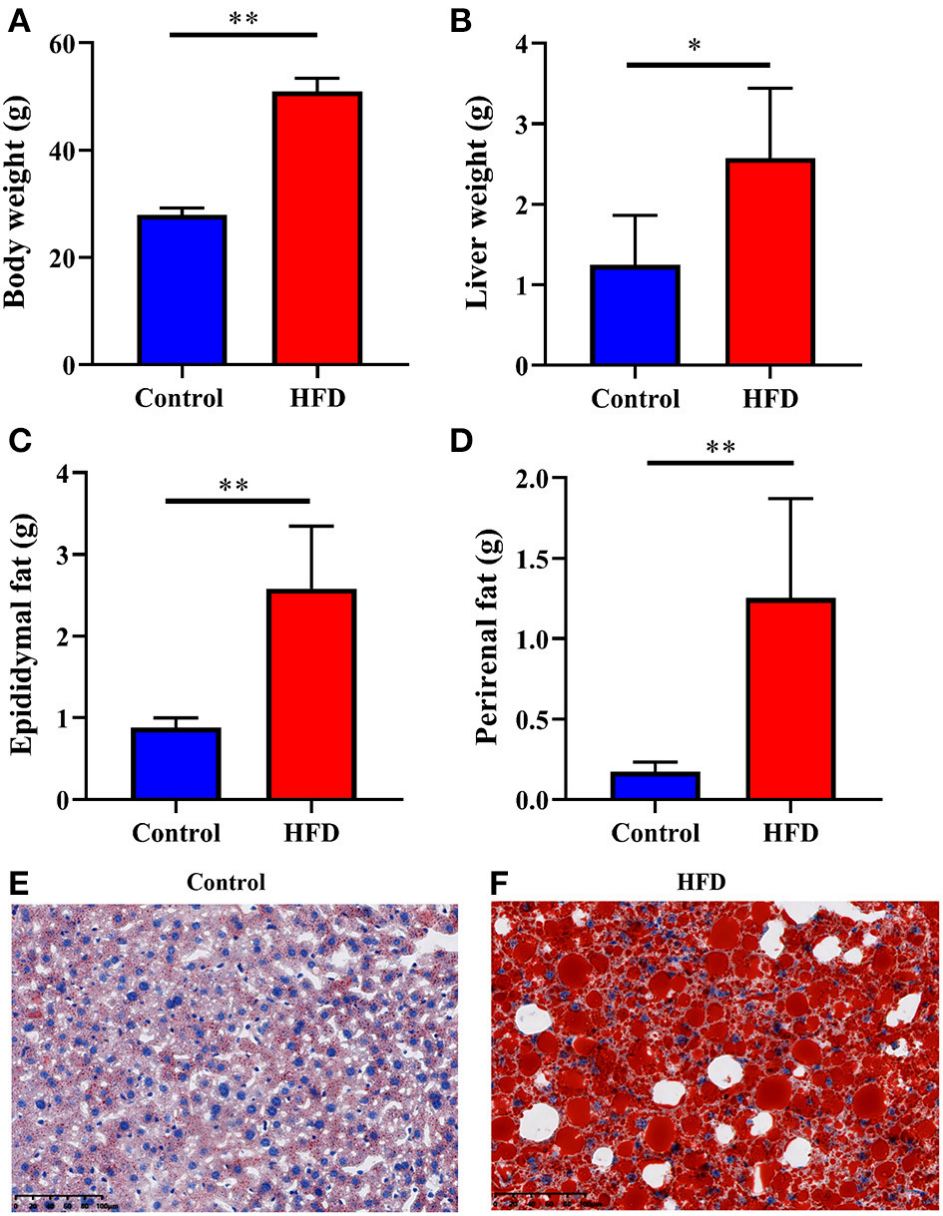
Characterization of the obesity induced by HFD. The Control and HFD groups mice were analyzed for body weight **(A)**, liver weight **(B)**, epididymal fat weight **(C)**, perirenal fat weight **(D)**. Oil-red O stains of paraformaldehyde-fixed liver sections prepared from the Control **(E)** and HFD **(F)** group mice. Data were expressed as mean ± SEM and statistically analyzed by using unpaired two-tailed Students’ *t*-test (*n* = 5). Asterisks (^*^ and ^**^) represent significant differences with *P* < 0.05 and *P* < 0.01, respectively.

To confirm whether obesity was induced by the HFD, we weighed each mouse, liver, epididymal fat, and perirenal fat individually in both of Control and HFD groups. The body weight, liver weight, epididymal fat, and perirenal fat in the HFD-treated mice were significantly higher than those of the Control group by 82.31% (*P* < 0.0001), 106.58% (*P* = 0.0234), 194.08% (*P* = 0.0011), and 627.91% (*P* = 0.0046), respectively (Figures 1A–D). To observe the fat content in the liver, we stained liver segments of mice in the Control and HFD groups. The number of lipid droplets in the liver of HFD group (Figure 1F) was obviously more and larger than that in Control group (Figure 1E), suggesting obesity had been induced by HFD.

### HFD Increased the Expression of ANGPTL4 in the Gastrointestinal Tract

**Figure 2:**
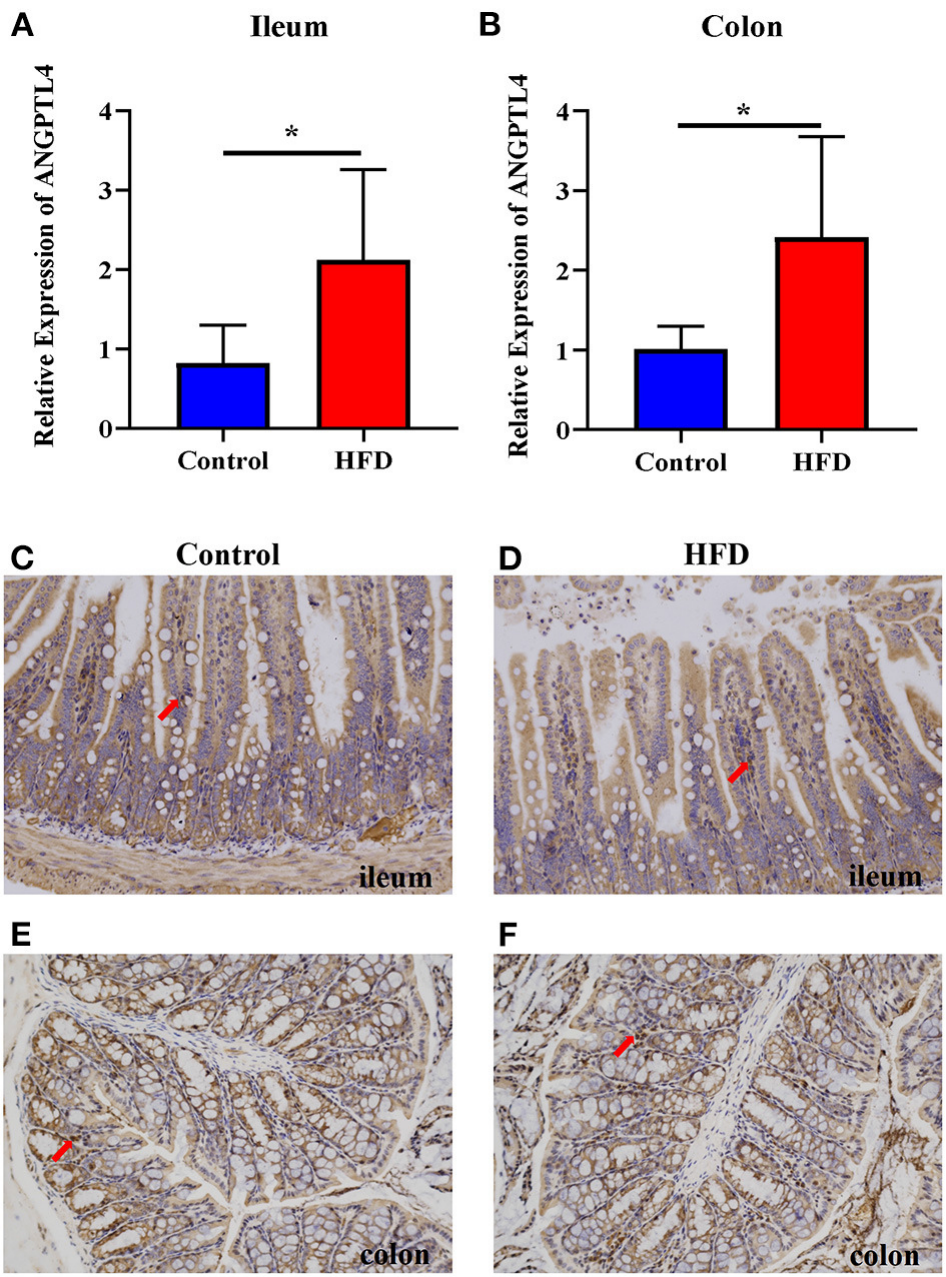
The expression of ANGPTL4 in the ileum and colon of the Control and HFD groups. The mRNA expression level of ANGPTL4 was measured in the ileum **(A)** and colon **(B)** of mice in the Control and HFD groups using RT-qPCR analysis followed by RNA isolation. Sections of the ileum **(C,D)** and colon **(E,F)** in Control group **(C,E)** and HFD group **(D,F)** were stained with rabbit polyclonal antibodies to mouse ANGPTL4. The arrowhead points in the direction where ANGPTL4-positive cells are present. Data were expressed as mean ± SEM and statistically analyzed by using unpaired two-tailed Students’ *t*-test (*n* = 5). Asterisks (^*^) represent significant differences with *P* < 0.05.

To examine whether the ANGPTL4 expression is involved in fat deposition, we determined the ANGPTL4 expression in the gastrointestinal tract by RT-qPCR analysis following RNA isolation and reverse transcription. Compared with the Control group, the relative expression of ANGPTL4 in the ileum (*P* = 0.0452) and colon (*P* = 0.0409) of mice in HFD group was increased significantly (Figures 2A,B). Furthermore, immunohistochemistry revealed the ANGPTL4-positive cells seemed to be more abundant in the HFD group (Figures 2D,F) than the Control group (Figures 2C,E), which was consistent with the trend of the relative expression of ANGPTL4 in the ileum and colon of mice in two groups.

### HFD Altered the Gut Microbiota

To investigate the structure of gut microbiota in mice of the Control and HFD groups, we collected the ileal and colonic contents from mice of the Control and HFD groups and analyzed the alpha -diversity of gut microbiota. The alpha-diversity indicated that the number of OTU, Shannon index, and Chao1 index in the Control group were significantly higher than that in the HFD group (*P* < 0.05) while the Simpson index was significantly lower of Control group compared to HFD group (Table 2).

**Table 2:** Indices of alpha-diversity.

**Gut**	**Item**	**Control**	**HFD**	**SEM**	***P*-value**
Ileum	OTU number	333^a^	218^b^	22.04	0.001
	Shannon index	3.26^a^	2.50^b^	0.18	0.022
	Simpson index	0.098^b^	0.196^a^	0.025	0.044
	Chao1 index	382^a^	248^b^	26.02	0.001
Colon	OTU number	379^a^	204^b^	29.86	<0.001
	Shannon index	4.38^a^	3.52^b^	0.16	0.001
	Simpson index	0.255^b^	0.643^a^	0.008	0.009
	Chao1 index	402^a^	225^b^	30.34	<0.001

*The different superscript letters in the same row represent a significant difference (p < 0.05)*.

**Figure 3:**
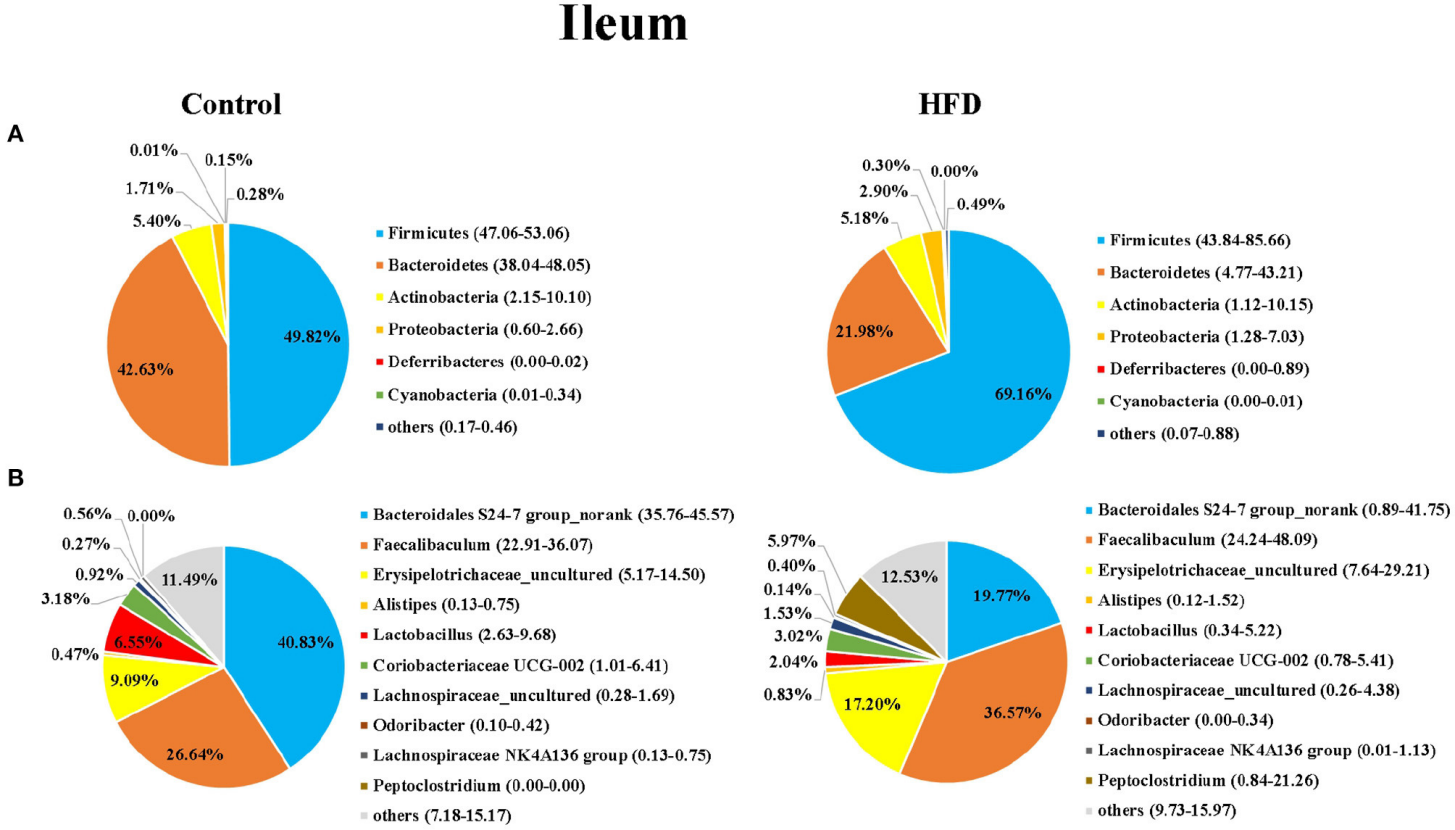
The bacterial community in ileum of the Control group and HFD group mice at the phylum **(A)** and genus **(B)** levels. The top 6 phyla and top 10 genera are shown.

**Figure 4:**
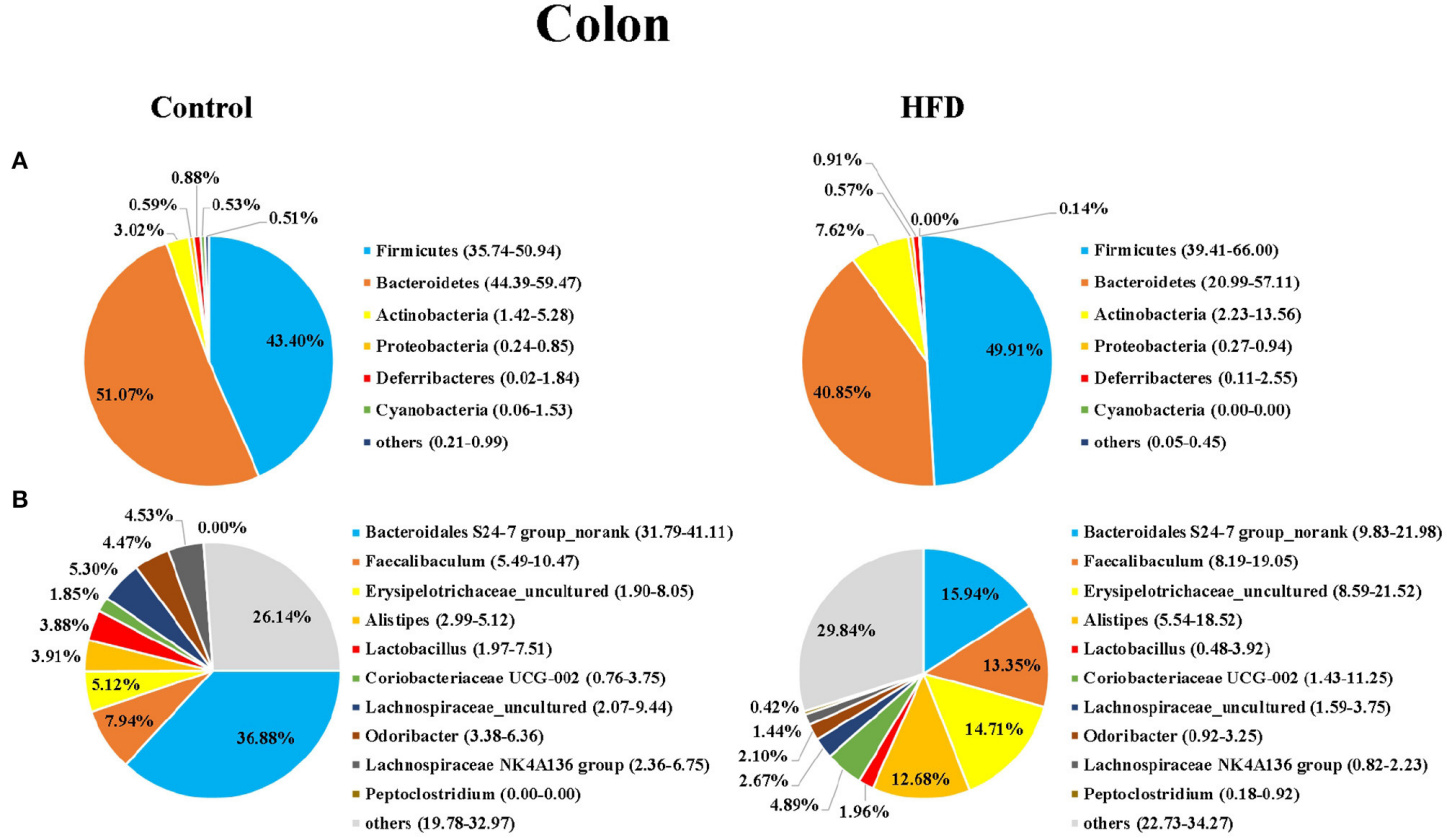
The bacterial community in colon of mice in the Control group and HFD group at the phylum **(A)** and genus **(B)** levels. The top 6 phyla and top 10 genera are shown.

Next, we analyzed the composition of gut microbiota in the ileum and colon of mice in the Control and HFD groups. Taxonomic analysis showed that the dominant bacteria phyla were Firmicutes, Bacteroidetes, Actinobacteria, Proteobacteria, Deferribacteres, and Cyanobacteria in both of ileum and colon, accounting for more than 99.12% of the total sequences in most samples (Figures 3A, 4A). The ratio of Firmicutes to Bacteroidetes, that was associated with the obesity phenotype, was remarkably higher in the ileum and colon of mice in the HFD group than those of the Control group (Figures 3, 4). Compared to the Control group, the relative abundance of Firmicutes in the HFD group was increased by 38.81 and 15.00% in ileum and colon (*P* < 0.05), respectively. However, the relative abundance of Bacteroidetes in the HFD group was decreased by 48.45 and 20.02% in ileal and colonic samples relative to the Control group (*P* < 0.05), respectively (Figures 3A, 4A). Cyanobacteria was less abundant in both of the ileum and colon of HFD group compared to Control group.

At the genus level, *Bacteroidales S24-7 group_norank, Faecalibaculum, Erysipelotrichaceae _uncultured, Alistipes, Lactobacillus, Coriobacteriaceae UCG-002, Lachnospiraceae_uncultured, Odoribacter, Lachnospiraceae NK4A136dium*, and *Peptoclostri* were the most abundant genera in both of the ileum and colon (Figures 3B, 4B). Compared to the Control group, the relative abundance of *Bacteroidales S24-7 group_norank* in the HFD group was decreased by 51.58% and 56.76% in ileum and colon (*P* < 0.05), respectively, while the relative abundance of *Faecalibaculum* was increased by 37.27 and 68.18% in the ileal and colonic samples (*P* < 0.05), respectively (Figures 3B, 4B). The relative abundance of *Lactobacillus* and *Odoribacter* was decreased while Alistipes and Peptoclostridium were increased in the ileum and colon (Figures 3B, 4B) of the HFD group as compared to the Control group.

**Figure 5:**
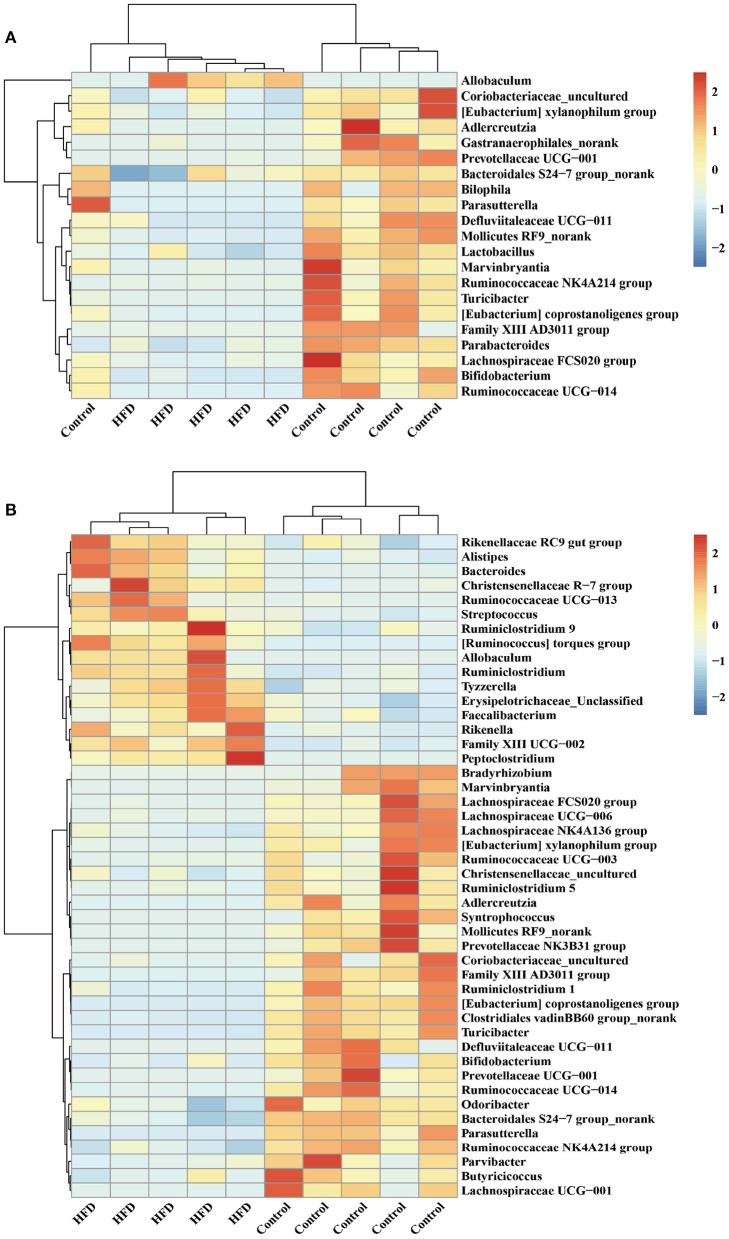
Hierarchically clustered heat map for the significantly different bacterial genera in ileum **(A)** and colon **(B)** of mice.

Hierarchical clustering together with a heat-map was performed to reveal the distinct characteristics of the significantly different bacterial genera (including the 21 from 248 total ileal bacterial genera, and 46 from 248 total colonic bacterial genera) based on the abundance of the identified bacterial genera in the Control group and HFD group (Figure 5). For example, the relative abundance of *Allobaculum* in ileum and colon of mice in the HFD group was significantly higher than that in the Control group (*P* < 0.05) (Figure 5). The relative abundance of *Adlercreutzia, Bifidobacterium, Prevotellaceae UCG-001* and *Ruminococcus* in the ileum and colon in HFD group was significantly lower than those in Control group (*P* < 0.05; Figure 5).

**Figure 6:**
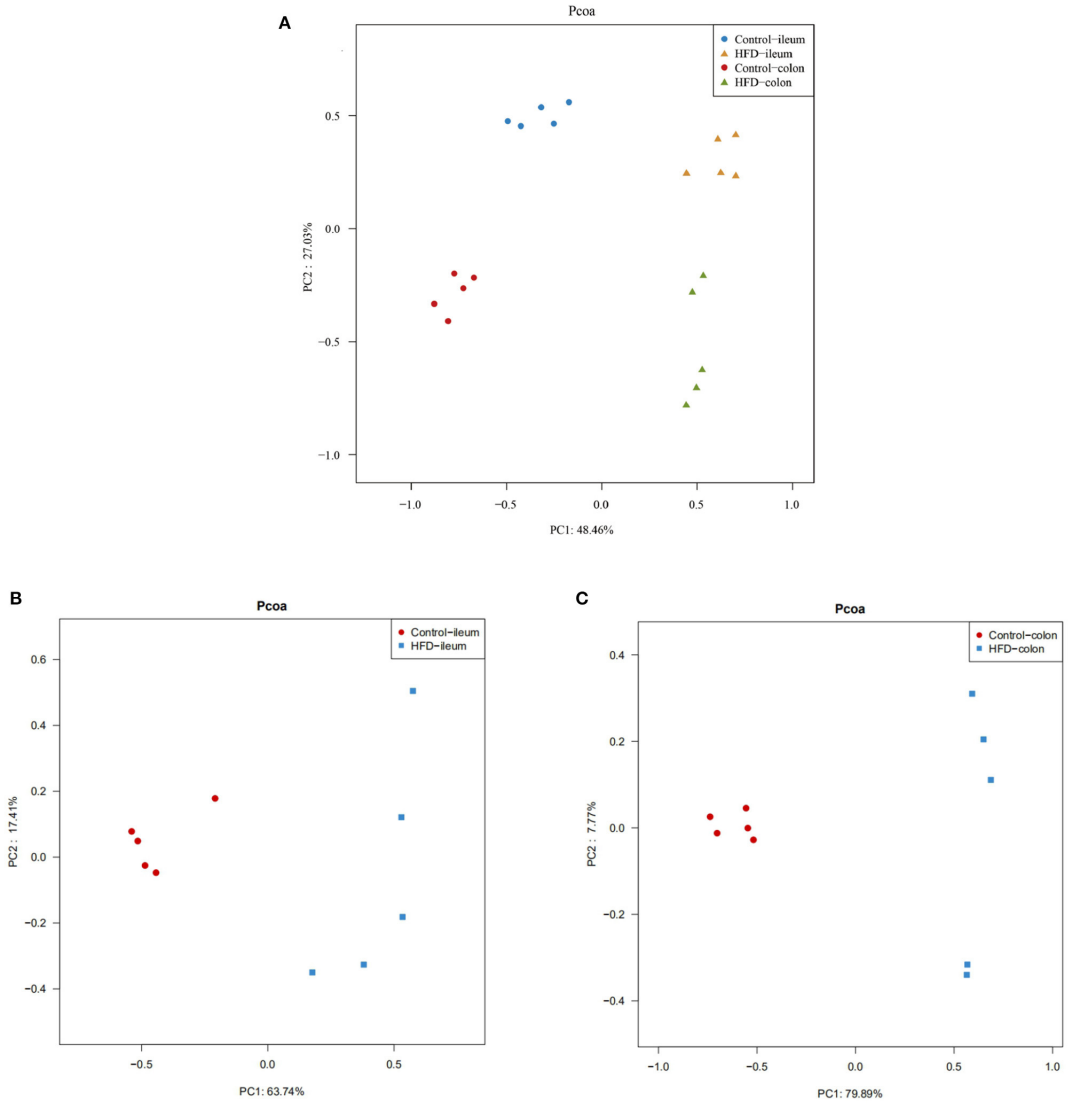
PCoA of the ileum and colon microbial community composition of the Control and HFD mice based on weighted unifrac distance. **(A)** The overall characteristics of all 20 samples were analyzed by a principal coordinate analysis. The differences in ileum **(B)** and colon **(C)** microbial community structure between the Control group and HFD group.

To evaluate the degree of discrepancy between the bacterial community structures of the Control group and HFD group mice, a principal coordinate analysis (PCoA) was performed. The ileal and colonic microbiota in the HFD group and the Control group were clearly separated from each other (Figure 6A). There was also a differently clustering of the bacterial community structure between the Control and HFD groups in ileum and colon (Figures 6B,C), indicating that the structure of the ileum and colon microbiota of the mice had a great difference between the Control and HFD groups.

### Correlation Between the Gut Microbiota and ANGPTL4 Expression

**Figure 7:**
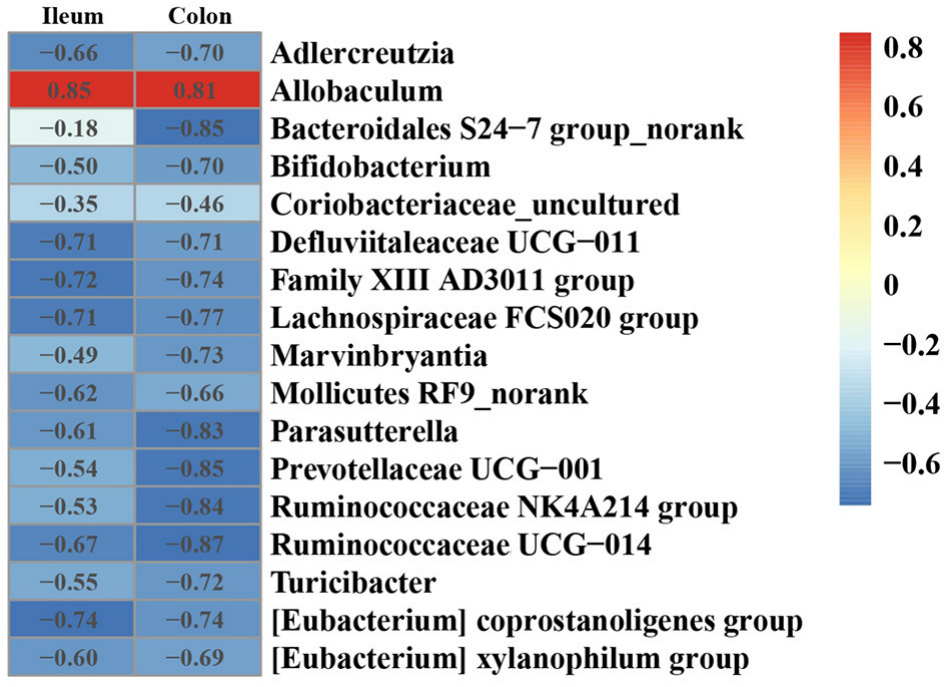
Heatmap of Spearman correlation between the relative expression of ANGPTL4 and microbial genus in ileum and colon of mice.

**Figure 8:**
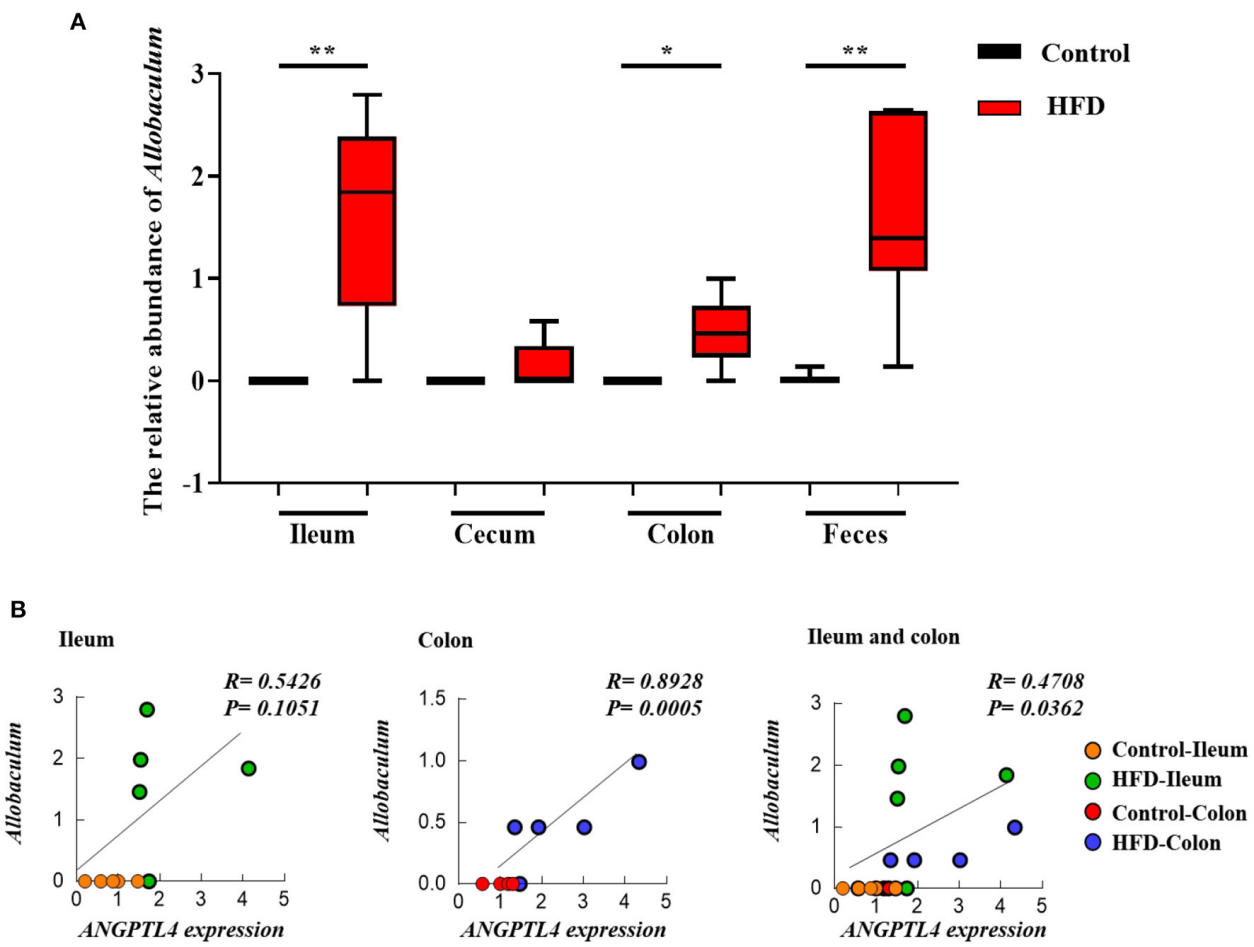
The relative abundance of *Allobaculum* in ileum, cecum, colon, and feces of Control group and HFD group mice **(A)**, and the correlation between the *Allobaculum* and ANGPTL4 expression **(B)** in ileum and colon. The data of cecum and feces were obtained from the NCBI database (DDBJ Accession Number: PRJDB7523 and NCBI Accession Number: SRP113647) **(A)**. Asterisks (^*^ and ^**^) represent significant differences with *P* < 0.05 and *P* < 0.01, respectively.

To investigate the effect of gut microbiota on the expression of ANGPTL4, the correlation analysis was conducted. The microorganism positively correlated with ANGPTL4 expression was *Allobaculum*, and others were negatively correlated with ANGPTL4 expression including *Adlercreutzia, Bacteroidales S24–7 group_norank, Bifidobacterium, Coriobacteriaceae_uncultured, Defluviitaleaceae UCG*−*011, Family XIII AD3011 group, Lachnospiraceae FCS020 group, Marvinbryantia, Mollicutes RF9_norank, Parasutterella, Prevotellaceae UCG*−*001, Ruminococcaceae NK4A214 group, Ruminococcaceae UCG*−*014, Turicibacter, (Eubacterium) coprostanoligenes group, (Eubacterium) xylanophilum group* (Figure 7). Combining with the raw data from other similar studies, it was found that the relative abundance of *Allobaculum* in feces, ileum, cecum, and colon was increased significantly in HFD group compared to Control group (Figure 8A). Correlation analysis confirmed the positive association between the abundance of *Allobaculum* and the expression of ANGPTL4 in the ileum and colon, wherein, the *Allobaculum* was much more relevant to the ANGPL4 expression in colon (*R* = 0.8928, *P* = 0.0005) (Figure 8B).

## Discussion

Obesity is one of the most important health topics worldwide. The incidence rate of obesity keeps increasing for many years due to the improvement of living standards, excessive calorie intake and lack of physical exercise (28). High-fat diet is the direct cause of obesity (6). Extensive researches showed ANGPTL4 was an important regulator of TG metabolism by inhibiting LPL and pancreatic lipase (29, 30). This study found that the body weight of the mice was increased by 82.31% (*P* < 0.05) with the HFD treatment. Compared to the Control group, the expression of ANGPTL4 in the ileum and colon of the HFD group was increased significantly. Similar studies showed that the expression of ANGPTL4 mRNA in small intestine (17) and liver (31) of mice was significantly increased with the HFD intervention. With the treatment of HFD, the absence of any regulation on TG digestion may lead to excess fatty acid uptake into enterocytes. The excess fatty acids would beyond the ability of re-esterification and chyle granule secretion, resulting in the lipid overload of intestinal cells. Therefore, the HFD regulated the expression of ANGPTL4 through a negative feedback mechanism to inhibit the excessive intake of TG. This feedback mechanism aimed to match the lipid uptake amounts of intestinal cells with the capacity for TG secretion. Similarly, the protection of ANGPTL4 against lipid overload in cardiomyocytes (32) and macrophages (18) had also been found. Additionally, Wostmann first discovered that the growth rate of GF mice was slower than normal mice. Gut microbiota also contributes to fat deposition (33). Bäckhed et al. (19) proved that gut microbiota could inhibit the expression of LPL cycle inhibitor, ANGPTL4, in the intestine. The ANGPTL4 expression level of GF mice were higher resulting in slower body weight gain of mice. The ANGPTL4 knock-out GF mice were more obese than the wild-type mice (34). These findings indicate that ANGPTL4 was a circulating medium between gut microbiota and fat deposition. However, the specific mechanism in the microbial regulation by ANGPTL4 expression is still unclear. Meantime, research on the modulation mechanism of ANGPTL4 expression have never stopped. Alex et al. (35) found that short chain fatty acids (SCFAs), as the main metabolites of gut microbiota, could effectively induce the expression of ANGPTL4 in human colon cancer cells T84 and HT29. Aronsson’s research showed that by being fed with the high-fat diet supplemented with Lactobacillus paraliquefaciens (F19), the body fat of mice was decreased significantly with the significant increase in the expression of ANGPTL4 (36). All of these prove that ANGPTL4 is involved in the regulation of gut microbiota on obesity.

Extensive studies have shown that the gut microbiota affects the body’s immune response (37), neural signal (38), and bone density (39), regulates intestinal endocrine function (40), provides energy (41), synthesizes vitamins (42) and many other compounds (43). Therefore, the changes of gut microbiota structure will induce gastrointestinal diseases even a series of diseases in other tissues or systems such as liver, heart, nervous system, respiratory system, and so on (44). In recent years, studies have found that gut microbiota are widely involved in host lipid metabolism and obesity (7, 45). By comparing the response of GF and normal mice to the HFD treatment, researchers found that GF mice had a resistance to HFD, but microbial remodeling led to obesity for GF mice (46, 47). Therefore, gut microbiota is an important factor regulating fat deposition. In this study, the relative abundance ratio of Firmicutes to Bacteroidetes was increased significantly in the ileum and colon of mice in the HFD group. Similar studies found that the relative abundance ratio of Firmicutes to Bacteroidetes in the gut microbiota of mice showed a downward trend through exercise (48). In the obese humans and mice, the relative abundance of Firmicutes and Bacteroidetes was improved and reduced, respectively (49, 50). These findings were consistent with the results of this experiment.

In order to further explore the effect of gut microbiota on the expression of ANGPTL4, we preformed a correlation analysis between the microbial community composition and ANGTPL4 mRNA level. The results showed that the expression of ANGPTL4 was positively correlated with the relative abundance of *Allobaculum*, but negatively correlated with *Adlercreutzia, Bifidobacterium, Prevotellaceae UCG-001, and Ruminococcus*. *Allobaculum* cells are rod-shaped, stain Gram-positive and are arranged in pairs or chains (51). Related research showed that *Allobaculum* could produce butyric acid, and positively correlated with ANGPTL4 expression (52, 53). Butyric acid could transactivate PPARγ and regulated ANGPTL4, the target gene of PPARγ, in colon cells (54). Early studies have shown that SCFAs play an important role in the human gastrointestinal system, especially in regulating the host fat storage (55, 56). Janssen found that ANGPTL4^−/−^ mice had significantly lower butyric acid levels than the wild-type mice. The butyrate-producing *Allobaculum* was less abundant in than the wild-type mice but *Adlercreutzia* abundance was significantly increased in ANGPTL4^−/−^ mice (52). Similar alteration in *Allobaculum* abundance was found in other studies (25, 26). The relative abundances of *Adlercreutzia* (26), *Bifidobacterium* (57), *Prevotellaceae UCG-001* and *Ruminococcus* (58) were significantly decreased in obese mice induced by HFD. These findings were consistent with the results of this study. On the other hand, studies revealed that the abundance of *Allobaculum* might be negatively correlated with inflammation, insulin resistance and obesity with the intervention of ginger (59), berberine and metformin (60), or prebiotics (61) in mice. Consistent with these studies, our results found the significant increase in the relative abundance of *Allobaculum* and the expression level of ANGPTL4 by the HFD treatment in mice. The increase in ANGPTL4 expression also had an inhibitory effect on the lipid absorption. These results indicate that *Allobaculum* could be a promising target for the strategy to control obesity.

## Conclusion

This study demonstrated that the HFD treatment significantly increased the expression of ANGPTL4 in the ileum and colon of mice with the enhancement of body weight, liver weight, epididymal fat weight, perirenal fat weight, and the lipid contents in the liver. This might be associated with the change in the composition of gut microbiota in mice including the significantly increased abundance of *Allobaculum* and the significantly decreased abundance of *Adlercreutzia, Bifidobacterium, Prevotellaceae UCG-001, and Ruminococcus* in the HFD mice. This work identified the positive correlation between the ANGPTL4 expression and the *Allobaculum* abundance and highlighted their important role in the regulation of lipid metabolism. This is meaningful to explore the regulation of ANGPTL4 by gut microbiota in the treatment of obesity.

## Data Availability Statement

The datasets presented in this study can be found in online repositories. The names of the repository/repositories and accession number(s) can be found below: https://www.ncbi.nlm.nih.gov/, PRJNA715938.

## Ethics Statement

The animal study was reviewed and approved by Institutional Animal Care and Use Committee of Zhejiang Academy of Agricultural Sciences.

## Author Contributions

ZZ and YX designed the experiment. ZZ, WL, SZ, XL, and YX conducted the animal experiments. ZZ, WL, HY, and YX wrote and revised the manuscript. ZZ, WL, YR, HY, and YX did experimental analysis, collected, and analyzed the data. All authors reviewed the manuscript.

## Conflict of Interest

The authors declare that the research was conducted in the absence of any commercial or financial relationships that could be construed as a potential conflict of interest.
